# Antimicrobial Solid Starch–Iodine Complex via Reactive Extrusion and Its Application in PLA-PBAT Blown Films

**DOI:** 10.3390/polym16111487

**Published:** 2024-05-24

**Authors:** Apoorva Kulkarni, Dimple Sharma, Alexander Ermlich, Shilpa Manjure, Ramani Narayan, Teresa M. Bergholz

**Affiliations:** 1 Department of Chemical Engineering & Material Science, Michigan State University, East Lansing, MI 48824, USA; narayan@msu.edu; 2 Saint Gobain Research North America, Northborough, MA 01532, USA; 3 Department of Food Science and Human Nutrition, Michigan State University, East Lansing, MI 48824, USA; 4 Northern Technologies International Corporation, Circle Pines, MN 55014, USA

**Keywords:** reactive extrusion, iodine, antibacterial, PLA, film, starch, PBAT

## Abstract

In this study, a solid masterbatch of starch–iodine complex with 6.7 wt.% iodine was prepared in pellet form using a ZSK-30 twin-screw extruder. Thermogravimetric (TGA) and isothermal TGA analysis of the pellets revealed that there was no significant loss of iodine due to sublimation during reactive extrusion. These solid pellets demonstrated antifungal properties when applied to strawberries via dip coating in an aqueous solution, extending their shelf life from two days to eight days, thereby reducing fungal growth and visual decay. Furthermore, the solid pellets displayed antibacterial activity against *E. coli*, as evidenced by the clear zone of inhibition observed in the Kirby–Bauer test. To enhance practical application, these pellets were further blended with PLA-PBAT film formulations at 10 and 18% by wt. to make blown films with effective iodine loadings of 0.7 and 1.3% by wt. These films showed superior antibacterial activity against *E. coli* compared with PLA control films and the commercial silver antimicrobial-containing films during direct inoculation tests as per ISO 22196. Tensile strength and elongation at break in machine direction (MD) for the starch–iodine-containing blown films were comparable to the control films in MD, but tensile strength was reduced to 37–40% in the transverse direction (TD). This was due to a non-uniform dispersion of the starch–iodine complex in the films, as confirmed by the visual and SEM analyses. Thus, this study illustrates the practical utility of the solid starch–iodine complex as a safe and efficient means of introducing iodine into an environment, mitigating the typical hazards associated with handling solid iodine.

## 1. Introduction

Nowadays, there is a noticeable surge in innovative active packaging materials aimed at enhancing safety in the food, healthcare, and personal care sectors. Increased awareness among consumers, particularly during the COVID-19 pandemic, has significantly driven the development of packaging materials to elevate safety standards [1]. One noteworthy development gaining traction is antimicrobial packaging, where an antimicrobial agent is integrated into the polymer packaging film to inhibit the activity of specific microorganisms [2]. Various natural and synthetic antimicrobial agents, such as bacteriocins, enzymes, metal ions, nanoparticles, essential oils and their extracts, organic acids, etc., are often incorporated into packaging systems [2,3]. Iodine is a well-known antimicrobial agent, used for more than a century in the pharmaceutical/medical industries [4,5]. It exhibits a wide range of antimicrobial effects, effectively targeting bacteria, mycobacteria, fungi, protozoa, and viruses. Additionally, it can be employed for the treatment of both acute and chronic wounds. Notably, a tincture of iodine, a blend of iodine and sodium/potassium iodide in water and ethanol, has been widely used for sterilization purposes. Various patents and papers also describe the incorporation of iodine into fabrics, polymers, and other materials for the sole purpose of its antibacterial and antiviral activity [6,7,8,9]. The main disadvantages associated with iodine are its instability and difficulty handling solid iodine. Iodine is also known for its toxicity at higher doses, and hence, it has found limited uses in applications outside the medical field (automotive, packaging, etc.) [10]. Iodine possesses a distinctive ability to form bonds with various polymeric materials [4]. Iodine can form complexes with several natural and synthetic polymers such as starch, cellulose, glycogen, chitosan, poly(vinylpyrrolidone) (PVP), etc. to form iodophors, which help to overcome difficulties associated with handling elemental iodine [7,8,11,12,13].

As a biobased, biodegradable material, starch is a naturally occurring resource that is biocompatible and safe for humans. Amylose in starch is known to form complexes with iodide polyanions (blue-colored complexes) [14]. Various studies for the structures of the amylose–iodine complex have shown that amylose exists in the form of a helical spiral with each turn of the helix containing six glucose units, and the iodine molecules get arranged along the spiral as triiodide ions [14,15,16]. Iodine is not very soluble in water. Iodine is dissolved in water in the presence of potassium iodide (KI) to form a linear triiodide (I_3_^−^) complex that is soluble in water. This triiodide molecule then slips up inside the hollow amylose helix. Water molecules interact with iodide to form a charge transfer complex, which causes the blue color of the complex. A recent publication by Pesek et al. (2024) provides a comprehensive analysis of the complex’s chemical composition [15]. Cross-linked starch iodine (CSI) has been studied for its antibacterial and antiviral activity [11,17]. In some studies, the addition of a povidone–iodine complex to polylactide (PLA) has resulted in composite materials with antifungal attributes [12]. López-Álvarez et al. (2022) also investigated the direct addition of iodine to polyamide (PA) and polyurethane (PU) matrices using a twin-screw extruder to generate polymer–iodine complexes [18]. To our knowledge, there have not been any studies to prepare and incorporate the starch–iodine complex in other polymer films or foams for antimicrobial packaging applications. 

Reactive extrusion has allowed mass production of thermoplastic starch (TPS) and maleated thermoplastic starch (MTPS) [19]. TPS and MTPS are often blended with other polymers like polylactide (PLA), polybutylene adipate terephthalate (PBAT), polycaprolactone (PCL), and glycol-modified polyethylene terephthalate (PETG) to improve their biobased content, certain performance properties like oxygen barrier or crystallization, biodegradation, etc., and to reduce the overall cost of the blends [19,20,21,22,23]. Iodine can be introduced during thermoplastic starch production in the extruder to make these thermoplastic starch–iodine complex pellets in solid form, which can be used as a vehicle to introduce iodine into an environment, bypassing the typical hazards associated with handling solid iodine. The potential applications for this innovative product could be diverse, including the following:

(1) Active packaging: as an additive for film to be used in active packaging, ensuring food safety.
(2) Air filtration systems: as an additive to make foams or filters to be used in air filtration systems for deactivating airborne viruses, including SARS-CoV-2.
(3) Polymer enhancement: in pellet form as an additive to provide antibacterial and antiviral properties in other plastics and polymers.

In the present study, a solid thermoplastic starch–iodine complex was prepared in the form of pellets in a ZSK 30 twin-screw extruder. These pellets were characterized for their iodine content and their antimicrobial activity. Further, this complex was added in various proportions to PLA-PBAT blown films, and the morphological, mechanical, thermal, and antibacterial properties of these films were evaluated. The choice of the PLA-PBAT system as a matrix was driven by its inherent sustainability, mechanical properties, and compatibility with diverse processing techniques. These properties make PLA-PBAT blends an attractive candidate for various applications, aligning with the objectives of our research to explore MTPS–iodine as an additive for various applications. The widespread adoption of PLA-PBAT systems in industries such as packaging and biomedical applications underscores their practical relevance and hence was selected as the matrix for our study. 

## 2. Experimental Section

### 2.1. Materials

High-amylose corn starch containing 12.8% moisture (*w*/*w*) was sourced from National Starch (Bridgewater, NJ, USA). Glycerol obtained from J.T. Baker (Phillipsburg, NJ, USA) was used as received. 2,5-bis(tert-butyl-2,5-dimethylhexane), 90% purity (known as Luperox 101), and maleic anhydride (MA) were procured from Sigma-Aldrich (Milwaukee, WI, USA). Molecular iodine and potassium iodide were obtained from Thermo Fisher Scientific (Waltham, MA, USA). Strawberries were bought from a local grocery store and were washed with distilled water and dried before use. The bacterial strain *E. coli* (TB0484) was used for bacterial cultivation and antimicrobial activity tests.

### 2.2. Preparation of MTPS–Iodine Pellets

The MTPS–iodine pellets were prepared in a co-rotating twin-screw CENTURY ZSK-30 extruder (Traverse City, MI, USA) using a process similar to that described in Kulkarni et al., 2021 [19]. The optimal quantities of iodine and potassium iodide were determined according to the calculations detailed in the Supplementary Materials. For 1 kg of total masterbatch, potassium iodide (26.9 g, 0.162 mol) was dissolved in 40 mL of water. In this solution, 53.2 g (0.419 mol) of solid iodine was added under stirring conditions. The result was a black/blue-colored solution of iodide anions in water.

**Figure 1:**
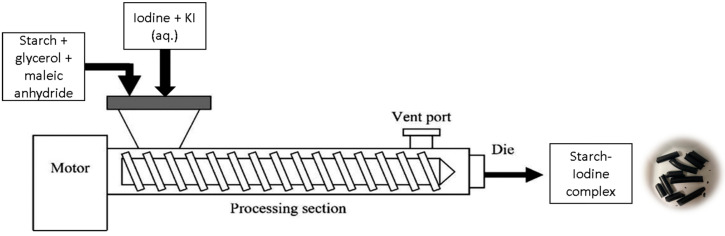
Production of MTPS–iodine pellets via reactive extrusion.

Next, pre-dried high-amylose corn starch (800 g) was mixed with glycerol (200 g) to make 1000 g of starch–glycerol mixture. To this mixture, 20 g of maleic anhydride and 2 g of Luperox 101 were added. Then the iodide solution was added to form a deep blue-colored powder. This powder was then extruded in a Century ZSK-30 co-rotating twin screw extruder using the temperature profile as shown in Table 1a. Other parameters such as screw speed, feed rate, and vent conditions mirrored those employed for MTPS, as explained in Kulkarni et al., 2021 [19]. The extrudate exiting the extruder underwent simultaneous air-cooling and pelletizing using a Scheer Bay pelletizer. The overall schematic for this process is shown in Figure 1.

### 2.3. Preparation of PLA-PBAT-Starch–Iodine Films

The MTPS–iodine masterbatch pellets were shipped to Natur-Tec^®^, a division of Northern Technologies Intl. Corp. (NTIC, Circle Pines, MN, USA), for the production of blown films. The MTPS–iodine pellets were mixed at 10 and 18% by wt. with commercial PLA-PBAT film formulations (detailed formulation not disclosed here) to yield films with 0.7% and 1.3% iodine in the resulting films. A LabTech LE20-30/C (Samutprakarn, Thailand) extruder with a 20 mm diameter screw with L:D of 30:1 and LabTech LF-250 blown film frame (Samutprakarn, Thailand) with a die diameter of 2 inches were used for making these films. The detailed temperature and processing conditions for making the films are shown in Table 1. Three different films with different thicknesses and iodine content were prepared as shown in Table 2, and their properties were compared with neat PLA-PBAT blown films and a commercial antimicrobial film supplied by Natur-Tec^®^ (Circle Pines, MN, USA) with silver as the antimicrobial agent.

## 3. Characterization and Analysis

### 3.1. Thermogravimetric Analysis (TGA)

The MTPS–iodine pellets, solid iodine, and neat MTPS pellets without any iodine were analyzed using TGA. TGA analysis was performed on all the samples under inert nitrogen using a TGA Q50 from TA Instruments (New Castle, DE, USA). The typical sample weight ranged from 5 to 7 mg. Each sample was placed in an aluminum pan and heated at a rate of 10 °C/min to a maximum temperature of 600 °C. The resulting weight loss (%) of the sample as a function of temperature (°C) was determined from this analysis. Then, to determine the iodine content in MTPS–iodine pellets, isothermal TGAs were also run on neat MTPS and MTPS–iodine samples. The sample was heated to 160 °C and maintained at that temperature for 30 min. Then it was heated further to 550 °C. The same was done with the control (neat MTPS) for comparison.

### 3.2. Antimicrobial Properties: Antifungal Test

As a simple qualitative way of testing the antifungal properties of the synthesized starch–iodine pellets, they were dissolved in water and applied to fresh strawberries as a coating [24]. The solution was prepared by dissolving 1 g and 2 g of starch–iodine pellets in water to yield 1% and 2% solutions, respectively. Fresh strawberries were obtained from the Meijer grocery store in East Lansing, MI, USA. The strawberries were washed carefully using distilled water and air-dried for 1 h before use. Then 15 strawberries were selected based on their uniform size, color, and absence of any physical or pathological damage. The fruits were evaluated for visual decay and weight loss. Then, five strawberries each were coated with 1% and 2% solutions of starch–iodine, and five were kept uncoated. All the strawberries were kept in a 75% relative humidity chamber for 11 days and were tested for visual decay, fungal growth, and weight loss immediately after coating (day 0) and at days 2, 4, 6, 8, and 11.

#### Visual Decay and Weight Loss of Strawberries

Photographs of the strawberries were taken every day and observed for signs of lesions, brown spots, or fungal growth. The weight loss of strawberries was expressed as a percentage loss based on the initial weight measured after coating [24]. Weight loss was calculated using the equation below:

$$Weight\;loss=\frac{initial\;weight-final\;weight\;}{initial\;weight\;}\times 100$$

### 3.3. Antimicrobial Properties

The antimicrobial properties were tested using the disk diffusion assay and direct inoculation assay following Shojaeiarani et al., 2020 [25]. *Escherichia coli* (*E. coli*) strain K-12 MG1655 was streaked onto Tryptic soy agar (TSA) media from a –80 °C freezer stock. One colony was picked and used to inoculate 5 mL of Tryptic Soy Broth (TSB). The bacteria were grown at 37 °C for 18 h.

#### 3.3.1. Antibacterial (Kirby–Bauer) Test

The disk diffusion test was performed on synthesized MTPS–iodine pellets with an iodine content of 6.7% by weight. A total of 100 mL of the 18 h broth culture was spread-plated on TSA plates, and a single iodine pellet was placed in the middle. These plates were incubated at 37 °C for 20 h. Three replicate plates were used for each pellet type. After 20 h, a clear zone of inhibition was indicative of the measure of antimicrobial property.

#### 3.3.2. Antimicrobial Properties: Antibacterial (Direct Inoculation) Test

The direct inoculation test was performed as per “ISO 22196-Measurement of antibacterial activity on plastics surfaces” [26]. PLA control films, silver antimicrobial film (AM Film), and the PLA films containing different concentrations of iodine were cut into sterile squares of 5 cm × 5 cm. The stomacher bag was cut at 4 cm × 4 cm, which was used to cover the films. A total of 2 mL of 18 h grown culture was centrifuged, the pellet was washed with 1X PBS (phosphate buffered saline) suspension buffer, centrifuged once again, and later suspended in 2 mL of 1X PBS, 100 μL of which was placed on top of every PLA film in a petri plate, covered with the stomacher bag film, and placed in the 37 °C incubator for 24 h. The enumeration of *E. coli* from PLA control films was taken at the time of inoculation and after 24 h, whereas samples from all the test films were taken after 24 h. To enumerate *E. coli*, the inoculated film sandwich was dropped with a set of sterile forceps in small sterile Whirlpak bags with 10 mL of 1X PBS buffer, homogenized at medium speed for 2 min in a Stomacher (Seward stomacher 400, VWR, Radnor, Pennsylvania, USA). Dilutions were plated on TSA plates. Three technical replicates were tested for all the experiments. At least three biological replicates and three technical replicates were tested for each film type. Statistical analyses, including one-way ANOVA and Tukey’s test, were performed using Microsoft Excel (Version 2404). 

### 3.4. Mechanical Properties

The tensile strength, tear strength, and penetration strength of PLA-PBAT control film supplied by Natur-Tec^®^ were compared with the MTPS–iodine-PLA-PBAT film samples SI1, SI2, and SI3. The samples were kept in a humidity chamber at 25 °C for 24 h before undergoing any analysis. Tensile testing was conducted using an Instron model 5544 (Instron, Norwood, MA, USA) equipped with a 100 N load cell and a grip separation speed of 20 inches/min following the ASTM D882-10 standard [27]. For each formulation, data from five samples was averaged and compared with the properties of control PLA-PBAT films. Additionally, tear strength testing was performed using a Thwing-Albert Elmendorf tear tester (West Berlin, NJ, USA) in accordance with the ASTM D1922 standard [28]. Five film samples were prepared and tested both in the machine direction (MD) and the transverse direction (TD) for each group.

### 3.5. Scanning Electron Microscopy

To investigate the dispersion of MTPS–iodine in PLA-PBAT films, a JOEL 6610 LV scanning electron microscope (manufactured by JEOL Ltd., Tokyo, Japan) was used. The fracture surfaces of all film samples were used for this analysis. Initially, the films underwent treatment with 6 N HCl for 24 h to eliminate the MTPS phase. Subsequently, they were air-dried for 12 h in a fume hood. Next, the films were immersed in liquid nitrogen for approximately 2 min and then fractured. The resulting fracture surfaces were affixed to aluminum stubs using high-vacuum carbon tabs. Finally, a gold coating was applied via a sputter coater, and the samples were examined using the JOEL microscope at 500× magnification and 10 kV.

## 4. Results and Discussion

### 4.1. Iodine Content Evaluation and Thermal Stability of the Blends

During the MTPS–iodine pellet preparation, iodine and KI were incorporated into the starch–glycerol mixture to achieve a total iodine content of 6.7%. However, given the potential for iodine sublimation at higher temperatures during extrusion, the final iodine content retained in the pellets after cooling was determined through thermogravimetric analysis (TGA).

**Figure 2:**
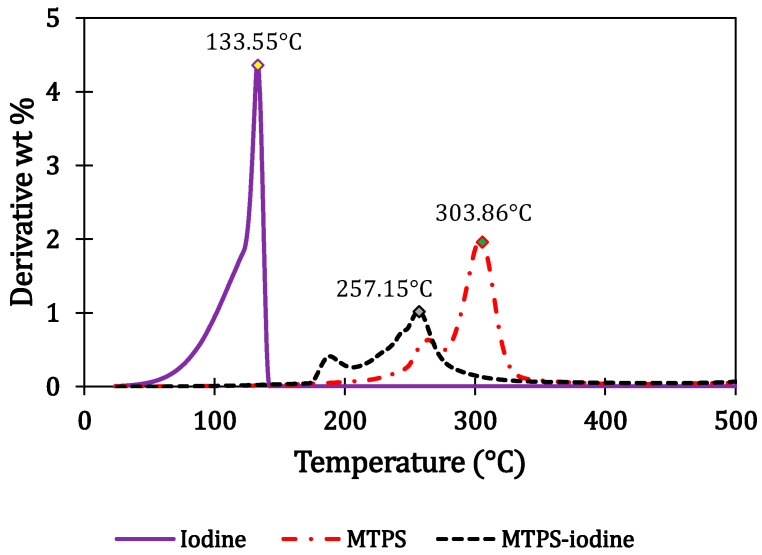
DTG curves for MTPS, MTPS–iodine, and iodine.

**Figure 3:**
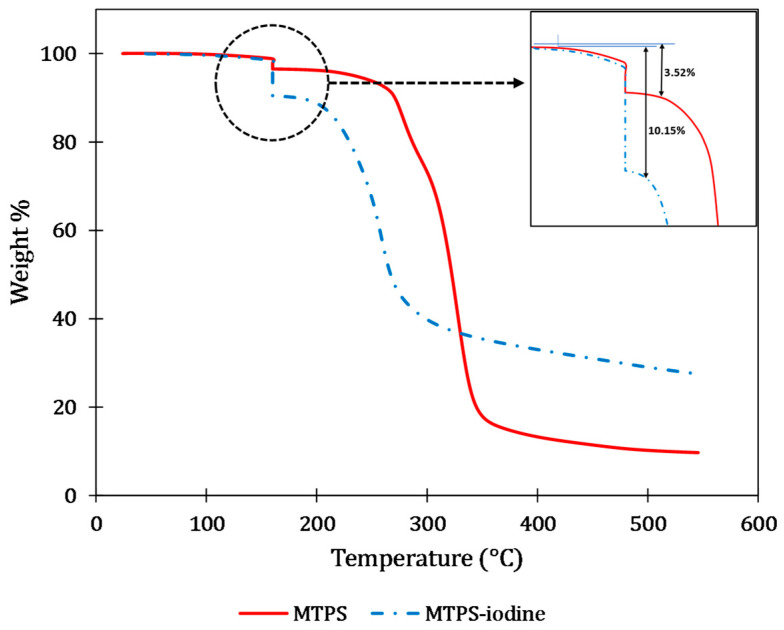
Isothermal TGA for MTPS and MTPS–iodine.

From the derivative thermogravimetric graphs (DTG) of neat MTPS (without iodine), iodine, and MTPS–iodine, the peak degradation temperatures for each of the compounds were determined (Figure 2). It was found that both MTPS (without iodine) and MTPS–iodine start degrading above 180–200 °C. In contrast, iodine alone degrades completely by 150 °C, with a peak degradation temperature of 133 °C. This suggested that the weight loss observed at 200 °C can be attributed to either moisture or the iodine present in the compound. Hence, to calculate the percentage of iodine present in the MTPS–iodine sample, it was heated to 160 °C and maintained at that temperature for 30 min to ensure complete degradation of iodine. Then it was heated further to 550° C. The same was done with the control (neat MTPS without iodine) for comparison. Then the difference between the weight loss for these two samples in the range of 25–200 °C was used to calculate the amount of iodine present in the MTPS–iodine pellet. This was done in triplicate to get an average iodine percentage. Figure 3 shows the isothermal TGA curves for MTPS and MTPS–iodine.

**Figure 4:**
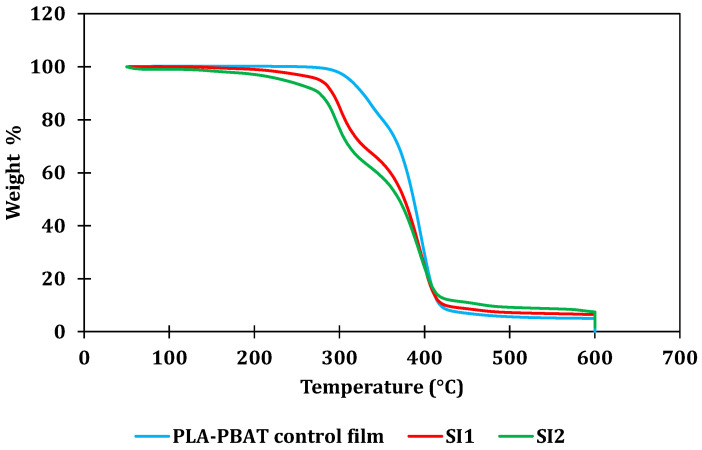
Comparative TGA plots for PLA-PBAT control film and SI1 and SI2 films containing starch–iodine complex.

The red curve in Figure 4 illustrates that for neat MTPS, there was an average weight loss of 3.52% between 25 and 200 °C. Given the absence of iodine in this compound, the 3.53% weight loss could be attributed to the presence of moisture in the sample. Conversely, in MTPS–iodine samples within the same temperature range, the average weight loss was measured at 10.18%. The difference in weight loss between these samples directly reflects the iodine content in the MTPS–iodine sample, calculated at 6.66%. The total iodine content added during pellet preparation was 6.7% by weight, closely matching the 6.66% iodine detected in the final pellets using TGA. This similarity suggested that negligible iodine loss occurred during the extrusion step. Our study provides robust evidence supporting the stability of iodine within the MTPS matrix, which holds significant implications for various applications. For instance, incorporating these pellets as additives into other plastics and polymers could offer valuable antibacterial and antiviral properties. Moreover, the demonstrated stability during extrusion processing assures the sustained efficiency of the pellets, ensuring their feasibility for widespread use across diverse industries. 

Another significant consideration for these formulations involves assessing the influence of adding the starch–iodine complex on the thermal stability of the films. A comparison of the thermal degradation temperatures between the control films and films incorporating 10% and 18% of the complex by weight was conducted using TGA, as depicted in Figure 4. The key findings from the TGA analysis are outlined in Table 3, including the temperature values associated with 5% and 50% weight loss, denoted as T5% and T50%, respectively. It is evident that the introduction of 10% and 18% starch–iodine complexes into the film compositions alters the onset degradation temperature T5% from 312.4 °C to 276.62 °C and 234.15 °C, respectively. The higher weight loss at lower temperatures could be due to the evaporation of moisture or low-molecular-weight compounds like glycerol. Likewise, a comparable trend is observed for the T50% temperature, which decreases from 386.9 °C for control films to 376.12 °C and 369.65 °C for SI1 and SI2 films, respectively. This behavior has been documented in prior studies by He et al. (2024) [29] and Chaves da Silva et al. (2017) [30], where the inclusion of starch led to a shift in thermal decomposition temperature towards lower values. This is attributed to the inherently lower thermal decomposition temperatures of starch when compared with PLA and PBAT.

### 4.2. Antifungal Properties: Effect on Weight Loss and Visual Decay

The weight loss and visual decay of strawberries coated with the starch–iodine solution were monitored every other day and compared with the control (uncoated) samples. Figure 4 shows the summary of the procedure and the results of this testing.

**Figure 5:**
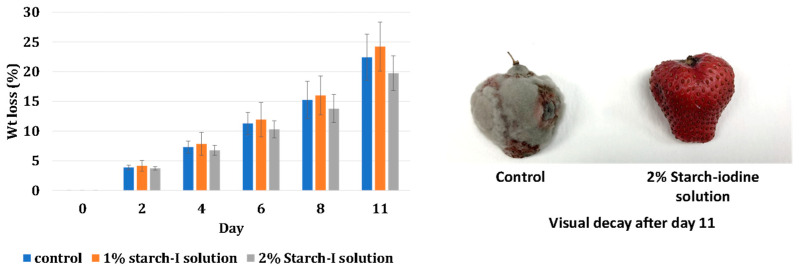
Weight loss and visual decay analysis for coated and uncoated strawberries.

The effect of coating the strawberries with a starch–iodine solution on weight loss is illustrated in the weight loss analysis graph (Figure 5). As expected, the weight loss increased during the storage period for all the treatments. The weight loss of the control and 1% starch–iodine and 2% starch–iodine-coated samples did not exhibit any significant difference (*p* > 0.05). The details on ANOVA analysis can be found in the Supplementary Materials Section.

The onset of visual fungal decay quickly manifested in the control samples by day 4 of storage. In contrast, coated samples delayed the onset of visual decay to day 6. For 2% starch–iodine-coated samples, the visual decay was even less, initiating on day 8 of the storage. These findings underline the potential of starch–iodine coating in reducing fungal growth and extending the visual freshness of strawberries over an extended storage duration. Salha et al. (2021), Khan et al. (2019), and Ait-boulahsen et al. (2018) [24,31,32] employed a comparable approach, utilizing chitosan-based and gelatin-based coatings to extend the shelf life of strawberries to approximately 13–15 days.

This initial test successfully served as a proof-of-concept for establishing the antifungal–antimicrobial properties of the starch–iodine pellets. However, in the case of its use for actual food applications, further detailed studies are necessary. These studies would assess the compatibility of this formulation with the human body, investigate the toxicity of iodine, and evaluate the edibility of this coating. While these aspects were not the focus of our current study, the concept of antifungal coatings extends beyond direct edible application. For instance, it can be used for cardboard boxes, wooden crates, paper bags, etc., which are used for packaging food items, and a coating with antifungal agents would enhance their durability and prevent decay. Additionally, antifungal coatings could also be used in building materials to prevent fungal infestations in humid environments. 

### 4.3. Antimicrobial Activity Studies on Pellets and Films

**Figure 6:**
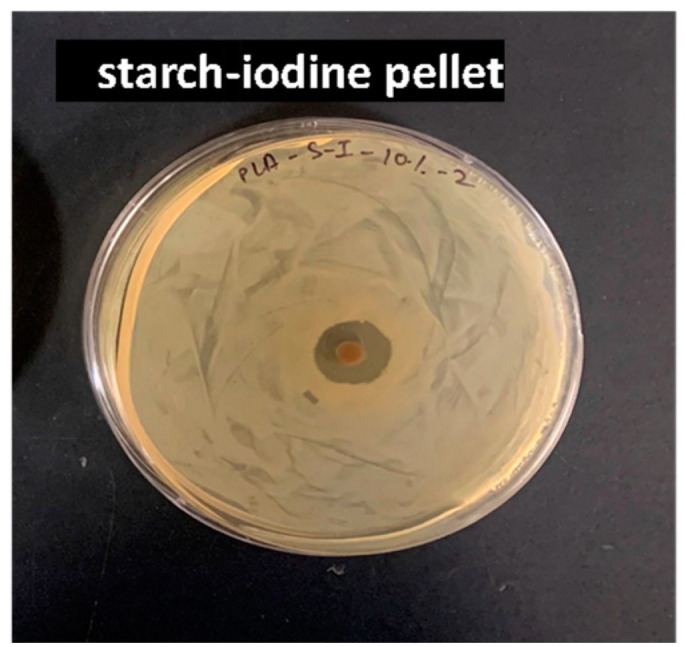
Photograph of the petri plate from disk diffusion; Kirby–Bauer test on starch–iodine pellets showing antibacterial zone of inhibition.

We conducted two distinct assays to assess the antimicrobial activity of starch–iodine pellets and the blown films. Initially, a disk diffusion method was employed for starch–iodine pellets and films. Notably, a clear zone of inhibition was observed in the bacterial lawn surrounding the pellets (Figure 6). For the films, there was no growth on the surface of the films, and no clear zone of inhibition was observed for them. It could be due to factors such as the diffusibility of iodine in the film.

**Figure 7:**
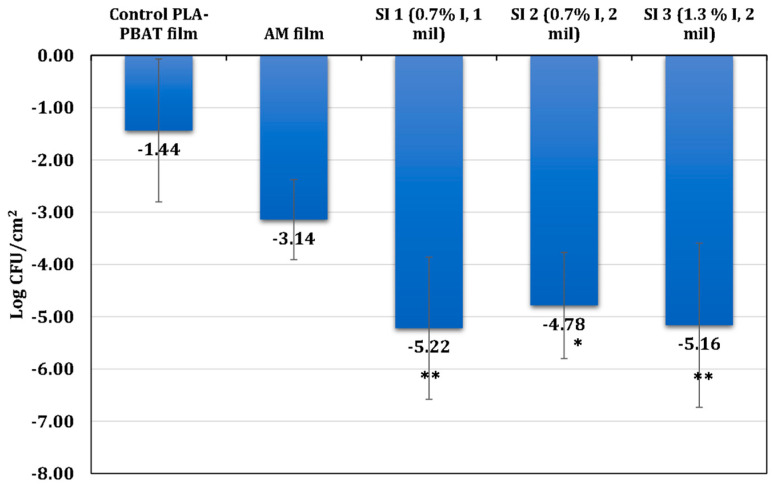
Average change in Log CFU/cm^2^ of the *Escherichia coli* (*E. coli*) K-12 strain after 24 h incubation on five different PLA film configurations (control PLA-PBAT; AM film; SI1, SI2, and SI3). The data is collected from four biological replicates for all films, except for three biological replicates for the AM film. Error bars for each film indicate the deviation from the standard mean of cell reduction from the time of inoculation on the control PLA-PBAT film. ANOVA showed significant differences for SI1 films (*p* = 0.006), S12 films (*p* = 0.016), and SI1.3 films (*p* = 0.007) compared with Control PLA-PBAT films. The asterisks represent the level of significance for each film compared with the control PLA-PBAT film.

In a separate approach, a slightly modified direct inoculation method, “ISO 22196-Measurement of antibacterial activity on plastics surfaces”, was used, where *E. coli* cells were directly inoculated on the surface of the films. The bacterial counts were observed on PLA-PBAT control and test films after 24 h of inoculation in comparison to the bacterial counts observed on the PLA control films at the time of inoculation. The differences in the average change in log CFU/cm^2^ were calculated. Figure 7 shows the average results for CFU/cm^2^ for all the samples.

As expected, the PLA/PBAT blend films without MTPS–iodine demonstrated no obvious antibacterial activity. This aligns with prior research on PLA-PBAT films, where the control films exhibited either no or very limited antimicrobial effects against *E. coli* [33,34,35]. It was observed that the survival of *E. coli* was negatively impacted in the presence of all the films (SI1, SI2, and SI3). The reduction was greater when compared with the control PLA-PBAT film and commercial antimicrobial film supplied by Natur-Tec^®^ (AM film) during 24 h incubation. These findings were derived from a one-way ANOVA and Tukey’s test, with further details available in Supplementary Materials.

### 4.4. Mechanical Properties and Morphology of the Films

**Figure 8:**
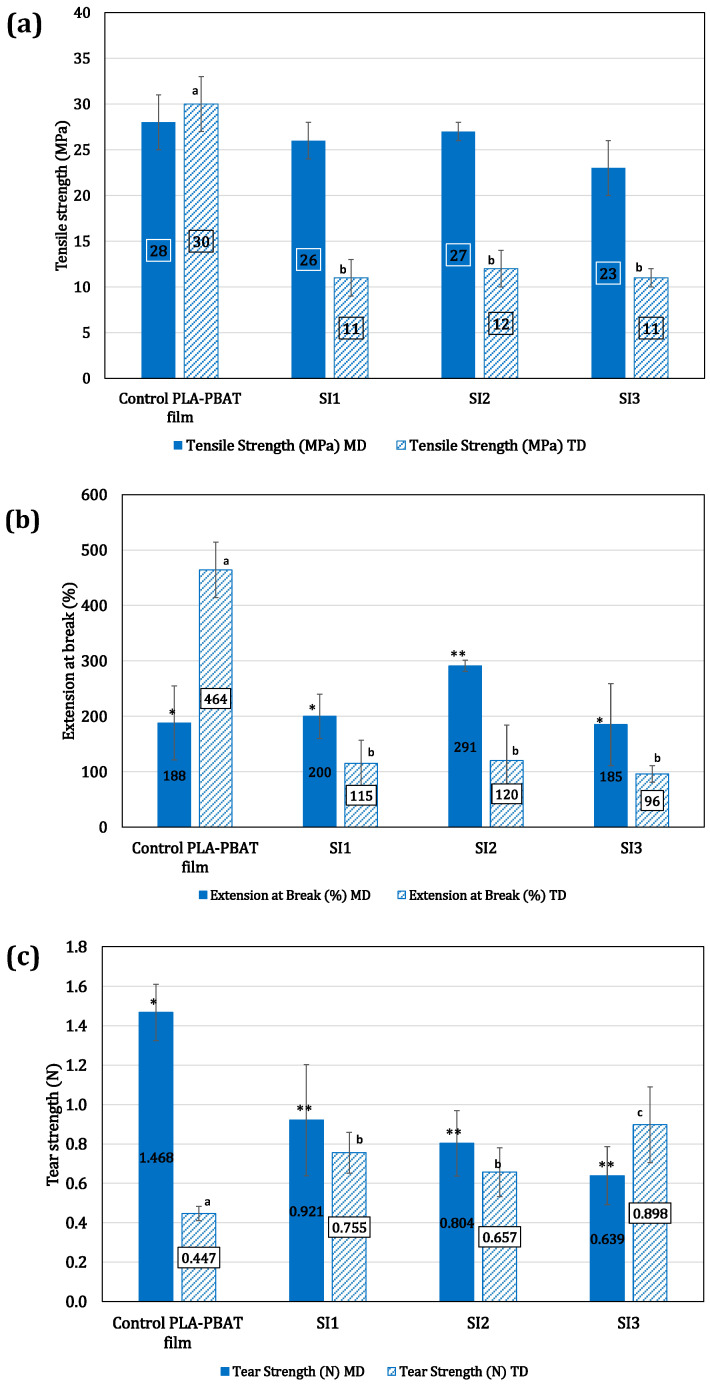
Mechanical properties for the different PLA-PBAT blown film formulations in machine and transverse direction: (**a**) tensile strength; (**b**) elongation at break; (**c**) tear strength. ANOVA showed significant differences in the SI1, SI2, and SI3 films compared with control PLA-PBAT films. The different letters and asterisks represent the level of significance for each film compared with the control PLA-PBAT film.

**Figure 9:**
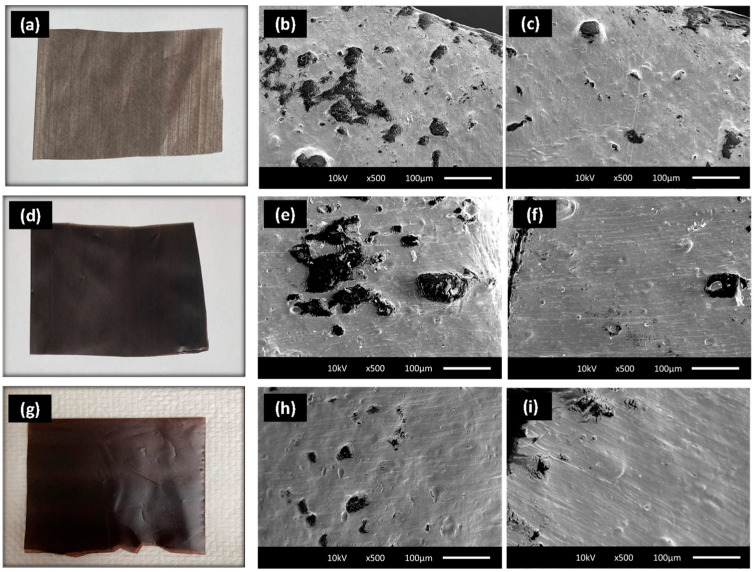
Visual and SEM images of the fractured surface of the films after selective extraction of the MTPS–iodine phase (**a**–**c**): SI1-0.7% iodine, 1 mil; (**d**–**f**): SI2-0.7% iodine, 2 mil; (**g**–**i**): 1.3% iodine, 2 mil.

Mechanical properties, including tensile strength and extension at break and tear strength, were measured for the PLA-PBAT–iodine complex films (SI1, SI2, and SI3), and their performance was benchmarked against the control PLA-PBAT films supplied by Natur-Tec^®^. ANOVA analysis showed that the tensile strength in machine direction (MD) for SI1 and SI2 was comparable to the control PLA films (Figure 8a). In TD, a significant reduction in the tensile strength and elongation at break were observed (Figure 8a). The same was observed for extension at the break in TD. The values for SI1, SI2, and SI3 were significantly lower compared with control PLA-PBAT films in TD. Table 4 shows the tensile strength extension and tear strength values for all the films, along with their ANOVA analysis. The detailed statistical analysis can be found in the Supplementary Materials Section. The asterics and different letters represent the level of significance for each film compared with the control. This observation can be linked to the poor dispersion of the MTPS–iodine complex within the PLA-PBAT blends utilized for blown films, as supported by morphological studies on the fractured surfaces of the films (Figure 9).

Figure 9 illustrates two distinct areas on the fractured surfaces of the films. For example, Figure 9b vs. Figure 9c. The cavities represent the MTPS–iodine phase that leached out with concentrated HCL. Notably, some regions displayed a high concentration of the MTPS–iodine phase, leading to the formation of large-size cavities indicative of agglomeration (Figure 9b,e,h). Conversely, areas with lower MTPS–iodine concentrations were observed in other regions (Figure 9c,f,i), affirming non-uniform distribution in the films. This agglomeration was more pronounced in SI1 and SI2. SI3 samples showed a comparatively smaller size of the agglomerates, and they were also distributed more uniformly compared with SI1 and SI2. This may be the reason that despite SI3 having almost double the MTPS–iodine content, its mechanical properties were comparable or even superior in certain aspects (e.g., tear strength in TD), underscoring the importance of uniform additive distribution in enhancing film properties.

This visual observation was corroborated by the films themselves. As shown in Figure 9a,d, the SI1 and SI2 have visual stripes in the films in some places, indicating a poor dispersion of the additive. For SI3, the run was smoother, and we could not see any visual difference in the dispersion of the additive (Figure 9g). Starch is hydrophilic, and polyesters are hydrophobic. So inherently, it is difficult to disperse starch or starch-based compounds in PLA or other hydrophobic polyesters. Consistent with our findings, several studies by Pan et al. (2016) [35], Raquez et al. (2008) [20], and Zhai et al. (2020) [23] have reported a similar trend. The addition of starch or thermoplastic starch (TPS) to PBAT or PLA matrices was observed to result in poor distribution and a reduction in mechanical properties. To address this issue, a potential strategy to enhance dispersion could involve the development of graft polyester, as studied by Hablot et al. [22]. In their work, they developed graft polyester by combining MTPS with PBAT. Further, they used this graft copolyester as a compatibilizer with neat PBAT, and the resulting mechanical properties of the blend and the dispersion improved significantly compared with the MTPS-PBAT blend without the graft polyester. We propose a similar approach in which the MTPS–iodine complex can be grafted on PBAT to create a grafted polyester. Subsequently, using this modified MTPS–iodine-PBAT as a compatibilizer with the blown film’s formulation (which predominantly consists of PLA and PBAT) could lead to improved overall performance. 

## 5. Conclusions and Future Work

We synthesized the solid MTPS–iodine complex using a ZSK-30 twin-screw extruder. The masterbatch pellets contained 6.7% of iodine by weight. TGA analysis of these pellets confirmed that there was no significant loss of iodine due to sublimation during the reactive extrusion process. Coating strawberries with this complex revealed its antifungal properties, while Kirby–Bauer tests showcased its antibacterial efficacy against *E. coli*.

These pellets were utilized as an antimicrobial additive in three PLA-PBAT blown films of varying thicknesses (1 mil and 2 mil) and iodine concentrations (0.7% and 1.3% by weight). Antimicrobial activity against *E. coli* was assessed using the ISO 22196 method. Films containing iodine exhibited significantly greater antimicrobial activity compared with control PLA films and commercial silver antimicrobial films.

Despite promising antimicrobial results, SEM imaging revealed poor dispersion of the MTPS–iodine complex in the blown films, leading to decreased tensile strength and elongation at break compared with control PLA-PBAT films. This may be attributed to the hydrophilic nature of starch, which poses challenges in blending with hydrophobic polymers like PLA and PBAT. Future research could explore graft copolyesters of MTPS–iodine and PBAT to enhance dispersion and improve film mechanical properties.

Beyond films, this versatile complex holds potential applications in air filtration systems or as a biobased additive for various plastics. Further research in these areas would contribute to establishing the versatility and effectiveness of this product across multiple fields. Further exploration into its efficacy against a broader range of pathogens could be useful in establishing the broad-spectrum efficiency of this complex.

## Figures and Tables

**Table 1:** (a) Temperature profile for making starch–iodine complex in Century ZSK-30 co-rotating twin screw extruder; (b) Temperature and processing conditions for making blown films on LabTech LE20-30/C extruder.

(a)									
**Feed Zone Temp (°C)**	**Zone 2 Temp (°C)**	**Zone 3 Temp (°C)**	**Zone 4 Temp (°C)**	**Zone 5 Temp (°C)**	**Zone 6 Temp (°C)**	**Zone 7 Temp** **(°C)**	**Zone 8 Temp** **(°C)**	**Zone 9 Temp** **(°C)**	**Die Temp** **(°C)**
60	80	100	110	120	120	130	140	140	130
(b)									
**Upper Die Temp (°C)**	**Lower Die Temp (°C)**	**Die Adapter Temp (°C)**	**Zone 3 (°C)**	**Zone 2 (°C)**	**Zone 1 (°C)**	**Screw RPM**	**Upper Pull Rate (ft/min)**	**Lower Pull Rate (ft/min)**	**Motor Amps**
170	170	165	160	154	148	100	10	10.5	77

**Table 2:** Sample name and composition.

Formulation Code	Iodine Content	Film Thickness
SI1	0.7%	1 mil (25.4 μm)
SI2	0.7%	2 mil (50.8 μm)
SI3	1.3%	2 mil (50.8 μm)
PLA-PBAT film	-	1 mil (25.4 μm)
AM film	Silver Antimicrobial present	1 mil (25.4 μm)

**Table 3:** Summary of the main results from thermal degradation of control PLA-PBAT films and SI1 and SI2 films containing starch–iodine complex.

Temperature (°C)		
	T5%	T50%
Control PLA-PBAT film	312.46	386.96
SI1 (10% MB)	276.62	376.12
SI2 (18% MB)	234.15	369.65

**Table 4:** Mechanical properties for the different PLA-PBAT blown film formulations in machine and transverse directions. Different letters in the superscript along a column indicate a significant difference between the values.

	**Tensile Strength (MPa)**		**Extension at Break (%)**		**Tear Strength (N)**	
	**Machine Direction (MD)**	**Transverse Direction (TD)**	**Machine Direction (MD)**	**Transverse Direction (TD)**	**Machine Direction (MD)**	**Transverse Direction (TD)**
Control PLA-PBAT	28 ± 3	30 ± 3 ^a^	188 ± 67 ^a^	464 ± 50 ^a^	1.47 ± 0.14 ^a^	0.45 ± 0.04 ^a^
SI1	26 ± 2	11 ± 2 ^b^	200 ± 40 ^a^	115 ± 42 ^b^	0.92 ± 0.28 ^b^	0.76 ± 0.10 ^b^
SI2	27 ± 1	12 ± 1 ^b^	291 ± 10 ^b^	120 ± 64 ^b^	0.80 ± 0.17 ^b^	0.66 ± 0.12 ^b^
SI3	23 ± 3	11 ± 1 ^b^	185 ± 74 ^a^	96 ± 15 ^b^	0.64 ± 0.15 ^b^	0.90 ± 0.19 ^c^

## Data Availability

The raw data supporting the conclusions of this article will be made available by the authors on request.

## Supplementary Materials

The following supporting information can be downloaded at: https://www.mdpi.com/article/10.3390/polym16111487/s1. S1: Calculation for iodine loading. S2: Antimicrobial activity for films. S3: Weight loss and visual decay. S4: Film mechanical properties.
