# Supplementary material for: Antimicrobial Solid Starch–Iodine Complex via Reactive Extrusion and Its Application in PLA-PBAT Blown Films
Source: Polymers (Basel). 2024 May 24;16(11):1487. doi: 10.3390/polym16111487 (PMC11175009; doi:10.3390/polym16111487)
Supplement: Supplementary file 1 [file polymers-16-01487-s001.zip › polymers-2969347-supplementary S1-Calculation for iodine loading .pdf]

S1 : Calculation for iodine loading

Based on several studies done on structure and chemistry of amylose-iodine complex, it has been postulated that amylose in the complex assumes a 6-fold helical conformation and the iodine molecule slips inside the amylose coil. Iodine, by itself is not very soluble in water. Hence KI is added. Together, they form polyiodide ions in the form I_n^-. These facts were used to calculate the total theoretical quantity of iodine that can be added to the high amylose starch used in this project.

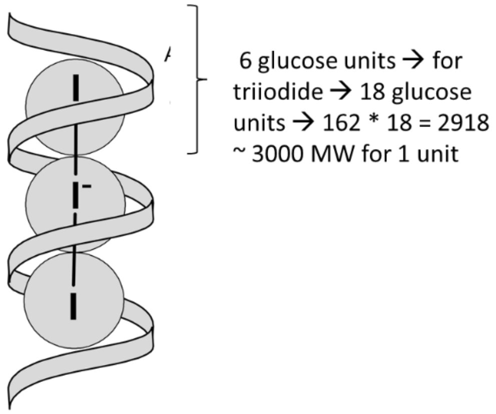

According to Baily et.al, 6 glucose units are required for 1 iodine atom.

So, for a tri-iodide ion, 18 glucose units are required

Molecular wt. for 1 anhydro glucose unit à 162 g/mol

Hence for 18 glucose units à 162* 18 = 2918 ~ 3000 g/mol

The high amylose starch used in this project has amylose content of 60-70% as given by the supplier.

An average of 65% amylose content was assumed for these calculations.

For making 1 kg of MTPS-iodine batch, the formulation was –

$800\ g\ starch\ +\ 200\ g\ glycerol\ +20g\ MA\ +\ catalyst$ (this amount has been optimized from our previous research Raquez et.al and Hablot et. al.)

$$out\ of\ 800g, 65\ \%\ is\ amylose$$

$$800 \times 0.65 = 520\ g\ amylose$$

$$1\ helix\ unit\ containing\ 1\ triiodide\ ion = 3000\ g/mol$$

$$hence, \frac{520}{3000} = 0.173\ moles\ of\ amylose\ helix\ units\ are\ present$$

$$for\ 1:1\ ratio\ of\ amylose\ helix\ unit\ to\ triiodide, 0.173\ moles\ of\ triiodide\ will\ be\ required$$

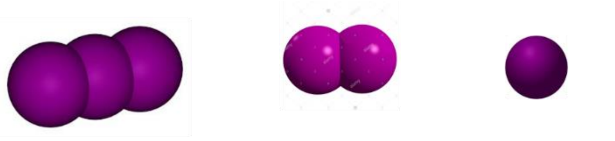

Triiodide I_3^- Mw = 380.71 | Iodine I_2 Mw = 253.8 | Iodide I^- Mw = 126.9

This corresponds to a total of 65. 74 g of triiodide (43.96 g of iodine, and 29.9 g of iodide).

$$\frac{65.7}{800} = 8.21\%\ by\ wt\ of\ starch$$

Assuming that there will be some loss in handling and processing, 10-12% extra iodine was added in the batch.
