# Supplementary material for: Antimicrobial Solid Starch–Iodine Complex via Reactive Extrusion and Its Application in PLA-PBAT Blown Films
Source: Polymers (Basel). 2024 May 24;16(11):1487. doi: 10.3390/polym16111487 (PMC11175009; doi:10.3390/polym16111487)
Supplement: Supplementary file 1 [file polymers-16-01487-s001.zip › polymers-2969347-supplementary S2-antimicrobial activity for films.pdf]

with 16 values

```
 summary(anova.model)
            Df Sum Sq Mean Sq F value  Pr(>F)
mydata$Group  4  40.05  10.012   5.221 0.00773 **
Residuals    15  28.76   1.918
---
Signif. codes:  0 ‘***’ 0.001 ‘**’ 0.01 ‘*’ 0.05 ‘.’ 0.1 ‘ ’ 1
> TukeyHSD(anova.model)
  Tukey multiple comparisons of means
    95% family-wise confidence level

Fit: aov(formula = mydata$Value ~ mydata$Group)
$`mydata$Group`
                                diff        lwr      upr     p adj
Iod-2-1.3%-Iod-0.7%-2mm   -0.3775825 -3.4011926 2.646028 0.9947947
PLA-0.7%-1mm-Iod-0.7%-2mm -0.4365300 -3.4601401 2.587080 0.9909643
PLA-silver-Iod-0.7%-2mm    0.9367625 -2.0868476 3.960373 0.8699730
PLA-T24-Iod-0.7%-2mm       3.3483165  0.3247064 6.371927 0.0267180
PLA-0.7%-1mm-Iod-2-1.3%   -0.0589475 -3.0825576 2.964663 0.9999966
PLA-silver-Iod-2-1.3%      1.3143450 -1.7092651 4.337955 0.6707268
PLA-T24-Iod-2-1.3%         3.7258990  0.7022889 6.749509 0.0127041
PLA-silver-PLA-0.7%-1mm    1.3732925 -1.6503176 4.396903 0.6353858
PLA-T24-PLA-0.7%-1mm       3.7848465  0.7612364 6.808457 0.0113042
PLA-T24-PLA-silver         2.4115540 -0.6120561 5.435164 0.1520265
```

with 15 values

```
summary(anova.model1)
             Df Sum Sq Mean Sq F value  Pr(>F)
mydata1$Group  4  42.32  10.580   6.493 0.00359 **
Residuals     14  22.81   1.629
---
Signif. codes:  0 ‘***’ 0.001 ‘**’ 0.01 ‘*’ 0.05 ‘.’ 0.1 ‘ ’ 1
 TukeyHSD(anova.model1)
  Tukey multiple comparisons of means
    95% family-wise confidence level

Fit: aov(formula = mydata1$Value ~ mydata1$Group)

$`mydata1$Group`
                                diff        lwr      upr     p adj
Iod-2-1.3%-Iod-0.7%-2mm   -0.3775825 -3.1901303 2.434965 0.9928508
PLA-0.7%-1mm-Iod-0.7%-2mm -0.4365300 -3.2490778 2.376018 0.9876564
PLA-silver-Iod-0.7%-2mm    1.6409492 -1.3969496 4.678848 0.4738877
PLA-T24-Iod- polymers-2969347-supplementary
PLA-0.7%-1mm-Iod-2-1.3%   -0.0589475 -2.8714953 2.753600 0.9999953
PLA-silver-Iod-2-1.3%      2.0185317 -1.0193671 5.056430 0.2851170
PLA-T24-Iod-2-1.3%         3.7258990  0.9133512 6.538447 0.0076418
PLA-silver-PLA-0.7%-1mm    2.0774792 -0.9604196 5.115378 0.2610494
PLA-T24-PLA-0.7%-1mm       3.7848465  0.9722987 6.597394 0.0067653
PLA-T24-PLA-silver         1.7073673 -1.3305315 4.745266 0.4368037
```
