# Supplementary material for: Antimicrobial Solid Starch–Iodine Complex via Reactive Extrusion and Its Application in PLA-PBAT Blown Films
Source: Polymers (Basel). 2024 May 24;16(11):1487. doi: 10.3390/polym16111487 (PMC11175009; doi:10.3390/polym16111487)
Supplement: Supplementary file 1 [file polymers-16-01487-s001.zip › polymers-2969347-supplementary S3 - weight loss and visual decay.pdf]

Day 2 No significant diff

ANOVA Table...

Source of Variation	Sum of Squares	d.f.	Variance	F	p
Between Groups:	0.4942	2	0.2471	0.7177	0.5077
Within Groups:	4.1313	12	0.3443		
Total:	4.6255	14			

Post-hoc tests...

```
Tukey HSD Post-hoc Test...
control vs 1%: Diff=0.2777, 95%CI=-0.7123 to 1.2678, p=0.7403
control vs 2%: Diff=-0.1618, 95%CI=-1.1518 to 0.8282, p=0.9013
1% vs 2%: Diff=-0.4395, 95%CI=-1.4296 to 0.5505, p=0.4841
```

day 4 No significant diff

ANOVA Table...

Source of Variation	Sum of Squares	d.f.	Variance	F	p
Between Groups:	2.9155	2	1.4578	0.8150	0.4657
Within Groups:	21.4648	12	1.7887		
Total:	24.3803	14			

Post-hoc tests...

```
Tukey HSD Post-hoc Test...
control vs 1%: Diff=0.5322, 95%CI=-1.7244 to 2.7889, p=0.8072
control vs 2%: Diff=-0.5476, 95%CI=-2.8043 to 1.7090, p=0.7973
1% vs 2%: Diff=-1.0799, 95%CI=-3.3365 to 1.1768, p=0.4340
```

Statistical analysis (anova and post-hoc test)

day 6 no significant diff

Compute

ANOVA Table...

Source of Variation	Sum of Squares	d.f.	Variance	F	p
Between Groups:	6.7674	2	3.3837	0.7264	0.5037
Within Groups:	55.8985	12	4.6582		
Total:	62.6659	14			

Post-hoc tests...

```
Tukey HSD Post-hoc Test...
control vs 1%: Diff=0.6552, 95%CI=-2.9865 to 4.2969, p=0.8820
control vs 2%: Diff=-0.9794, 95%CI=-4.6211 to 2.6623, p=0.7580
1% vs 2%: Diff=-1.6346, 95%CI=-5.2763 to 2.0071, p=0.4769
```

day 8 no signifiant difference

ANOVA Table...

Source of Variation	Sum of Squares	d.f.	Variance	F	p
Between Groups:	12.7141	2	6.3571	0.7345	0.5001
Within Groups:	103.8649	12	8.6554		
Total:	116.5790	14			

Post-hoc tests...

```
Tukey HSD Post-hoc Test...
control vs 1%: Diff=0.7545, 95%CI=-4.2096 to 5.7186, p=0.9140
control vs 2%: Diff=-1.4632, 95%CI=-6.4273 to 3.5009, p=0.7181
1% vs 2%: Diff=-2.2177, 95%CI=-7.1818 to 2.7464, p=0.4800
```

day 11 no significant diff

Compute

ANOVA Table...

Source of Variation	Sum of Squares	d.f.	Variance	F	p
Between Groups:	51.0060	2	25.5030	1.8809	0.1947
Within Groups:	162.7103	12	13.5592		
Total:	213.7163	14			

Post-hoc tests...

```
Tukey HSD Post-hoc Test...
control vs 1%: Diff=1.8076, 95%CI=-4.4055 to 8.0208, p=0.7241
control vs 2%: Diff=-2.6810, 95%CI=-8.8942 to 3.5321, p=0.5028
1% vs 2%: Diff=-4.4887, 95%CI=-10.7018 to 1.7245, p=0.1734
```
