# Supplementary material for: Antimicrobial Solid Starch–Iodine Complex via Reactive Extrusion and Its Application in PLA-PBAT Blown Films
Source: Polymers (Basel). 2024 May 24;16(11):1487. doi: 10.3390/polym16111487 (PMC11175009; doi:10.3390/polym16111487)
Supplement: Supplementary file 1 [file polymers-16-01487-s001.zip › polymers-2969347-supplementary S4 - film mechanical properties .pdf]

Natur-Tec®

Film Results Summary

	Campaign #	Lab Line			
		Control PLA-PBAT film	SI1	SI2	SI3
SPECIFICATIONS	**Thickness (mil)**	**1.00**	**1.00**	**2.00**	**2.00**
**Tensile Strength (MPa) MD**	**Average**	28	26	27	23
	**Std Dev**	3	2	1	3
**Extension at Break (%) MD**	**Average**	188	200	291	185
	**Std Dev**	67	40	10	74
**Tensile Strength (MPa) TD**	**Average**	30	11	12	11
	**Std Dev**	3	2	2	1
**Extension at Break (%) TD**	**Average**	464	115	120	96
	**Std Dev**	50	42	64	15
**Tear Strength (N) MD**	**Average**	1.468	0.921	0.804	0.639
	**Std. Deviation**	0.143	0.282	0.166	0.147
**Tear Strength (N) TD**	**Std. Deviation**	0.036	0.103	0.124	0.193
